# Intracavity Laser Wavelength Tuning by a Programmable Forward-Only Silicon Photonics Interferometer Mesh

**DOI:** 10.1021/acsphotonics.6c00194

**Published:** 2026-07-30

**Authors:** Marek Vlk, Carson G. Valdez, Anne R. Kroo, Charles Roques-Carmes, Shanhui Fan, David A. B. Miller, Olav Solgaard

**Affiliations:** † Department of Physics and Technology, 8016 UiT The Arctic University of Norway, P.O. Box 6050 Stakkevollan, Tromsø NO-9037, Norway; ‡ 6429 Stanford University, Ginzton Laboratory, 348 Via Pueblo Mall, Stanford, California 94305, United States

**Keywords:** compact tunable laser, photonic integrated circuit, silicon photonics, optical filter, erbium laser, interferometer mesh, programmable photonics

## Abstract

We exploit a recently proposed concept for a programmable, mechanically robust, and tunable nonresonant silicon photonic integrated circuit filter and experimentally demonstrate it for tuning an erbium-doped fiber laser ring cavity. This filter integrates forward-only interferometer meshes together with a waveguide delay line array for fully programmable and tunable filtering. Unlike more popular microresonator-based filters, this approach offers transmission function engineering, broad free spectral range and smooth tuning without matching individual resonances, and extension to multiwavelength operation. Moreover, this filter can be readily fabricated in a commercial foundry with standard dispersive components. For a first experimental verification, we designed a filter with a 100 GHz (∼0.8 nm) free spectral range and a 25 GHz (∼0.2 nm) bandpass width at center wavelengths around 1550 nm. We show numerically that dispersion effects are negligible across >40 nm bandwidth aside from grating coupler loss. Experimentally, we confirm single-wavelength laser operation with a continuous tuning range of ∼0.6 nm around 1555 nm and output power up to 25 mW.

## Graphical abstract

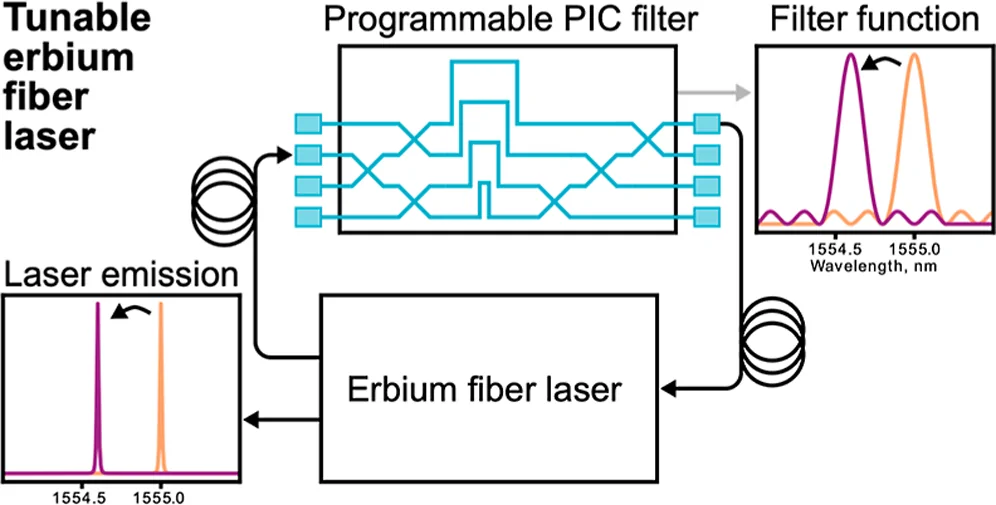

## 1 Introduction

Tunable lasers are used in many important applications including spectroscopy,^1–7^ LIDAR,^8–10^ and optical communication.^11–13^ The typical tunable laser comprises a gain medium, a resonator, and a tunable wavelength filter that determines the laser output wavelength. Traditionally, they have relied on moveable prisms or gratings for their dispersion to select the resonant wavelength, an approach still used in external cavity lasers today.^14^ Nevertheless, compact solid-state devices have emerged with the advent of wavelength division multiplexing (WDM) in optical communications.^12,15^ Wavelength tuning over 6–7 nm near 1550 nm has been achieved by a tunable distributed Bragg reflector (DBR) and phase shifter integrated with the active gain medium in a single semiconductor. All control is achieved by carrier injection into the different sections of the semiconductor. This changes the semiconductor refractive index, modulating the phase shift and the DBR reflection peak wavelength. However, the achievable wavelength shift is limited by the optical loss from free carrier absorption.^16^ Wider tuning has been obtained with sampled grating DBRs having periodic reflection spectra in contrast to a single reflection peak of a DBR. Lasers with two such gratings can have an effective free spectral range (FSR) over tens of nanometers. This so-called Vernier configuration utilizes gratings with different FSRs and allows resonance only where the reflection peaks of the two gratings are aligned. Such lasers have achieved tuning over 40 nm.^17^ Similar capability has been obtained more recently with nanophotonic low-loss microresonators, enforcing optical feedback at their sharp resonances. Microresonators, usually shaped as rings or racetracks, can be both placed externally, which is typical for frequency stabilization, or embedded in the laser cavity. A single microresonator is suitable for laser frequency stabilization,^18,19^ but not for wavelength tuning due to its low-nanometer-range FSR. Wide tuning, on the other hand, is again attained in Vernier configurations with two or more coupled microresonators,^13,19–21^ or by coupling multiple microresonators to a common bus waveguide.^22^ These lasers unite gain media with photonic integrated circuits (PICs) offering flexible operation with purely electronic control and without the need for moving parts or bulk optics.

Although such compact lasers now tune across several tens of nanometers, smooth and fast wavelength tuning requires complex mapping and matching of resonances.^21^ This can be avoided with arrayed waveguide gratings (AWGs) used in WDM applications.^23^ AWGs split light into a set of delay lines, the arrayed waveguides, and interfere the time-delayed fields resulting in periodic bandpass function that depends on the number of delay lines and the delay line increment. Up to *N* separate spectral channels, each being a single bandpass function, can be obtained with *N* delay lines. Nevertheless, as most research into compact tunable lasers has narrowed to the integrated gratings and microresonators, AWGs have been sidelined as elements for multiplexing or switch fabrics.^12^ They are typically passive devices with fixed spectral response and stringent fabrication tolerances, making them unfavorable in tunable filter architectures; An AWG must deliver precise time delays from the arrayed waveguides and split and interfere the optical fields with minimal coupling loss in integrated passive beam splitters. However, these limitations can be mitigated after fabrication with phase shifters.

Here, we demonstrate intracavity laser wavelength tuning with a new photonic integrated circuit filter (PICF), which integrates programmable interferometer meshes and a waveguide delay line array,^24,25^ a component similar to AWGs. The meshes, which are cascades of programmable 2 × 2 nodes,^26^ replace the conventional beamsplitters in AWGs; they split the input field among the waveguide delay lines and interfere the time-delayed outputs of the delay line array. Their programmability compensates fabrication imperfections, and, crucially, enables tunable filter functions as was recently demonstrated.^25^ We realized the new PICF in commercial foundry silicon photonics for operation at telecom wavelengths around 1550 nm. This is compatible with the gain bandwidth of erbium-doped fibers, spanning multiple optical communication bands with typical maximum in the C-band (1530–1565 nm). The use of PICFs is particularly practical for lasers with fiber gain media, because the PICF can be directly coupled to the fiber gain medium with an interface that is optically and mechanically robust. The PICF in this work can be adapted to other wavelength bands and integrated with thin-film or semiconductor gain media. Moreover, its layout can be altered for multiwavelength lasing with individually controlled emission wavelengths.

To verify the laser wavelength tuning capability, we built a test erbium fiber laser in a ring cavity configuration shown in Figure 1. The PICF is included in the ring cavity path such that it modulates the transmission profile by imposing its preprogrammed bandpass function. The bandpass position is then shifted by changing the PICF programming, which directly drives the laser emission wavelength. For this experimental verification, we opted for a PICF with four delay lines giving a 100 GHz FSR (or ∼0.8 nm) and 25 GHz bandpass width (or ∼0.2 nm). In our proof-of-principle experiments, we demonstrate laser operation with a wavelength tuning range of ∼0.6 nm around 1550 nm with output power near 25 mW. The linewidth is currently limited to 2 GHz due to high roundtrip loss and the 25 GHz broad bandpass function, but it could be reduced with further integration and more suitable PICF designs. We verify numerically that the dispersion of standard silicon photonics is insignificant across the 100 GHz FSR, but the power transmission is restricted by grating couplers to a 1 dB bandwidth of ∼14 nm. Without the grating couplers, the dispersion is negligible over more than 40 nm bandwidth. Finally, we discuss how to tailor such PICFs for wider FSRs and narrower bandpass functions.

**Figure 1:**
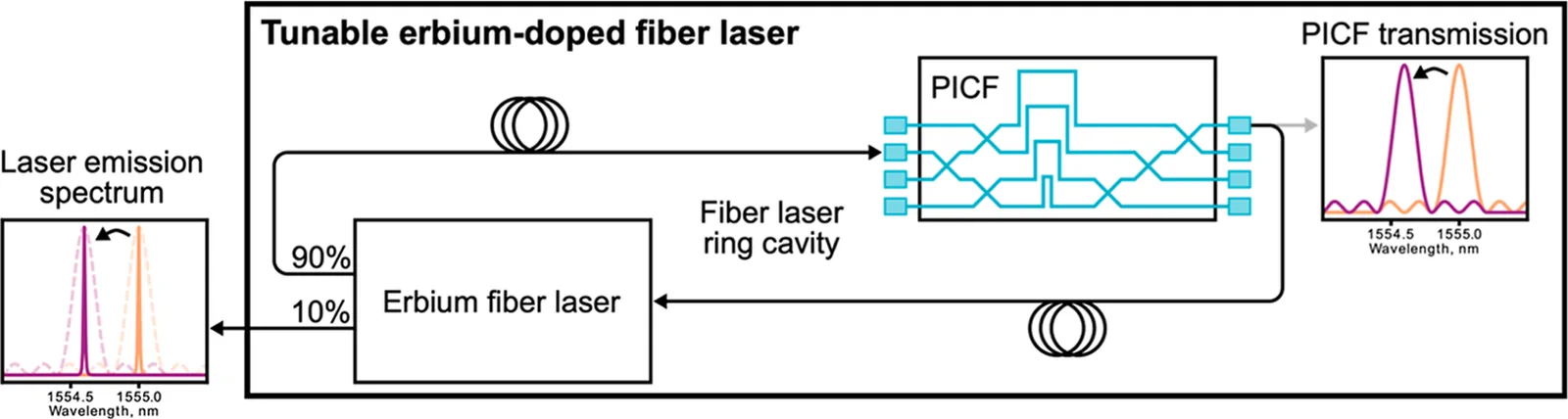
Schematics of the tunable laser ring cavity with a 4 × 4 programmable PICF. The programmable PICF is included in the laser cavity and modulates the cavity transmission with a preprogrammed filter function (right). The filter function is electronically shifted, and the laser emission wavelength (left) follows the filter as indicated. Black solid lines represent single-mode optical fibers, and blue solid lines represent single-mode optical waveguides. The optical fibers and waveguides are interfaced with grating couplers.

### 1.1 Programmable 4 × 4 PICF

Our programmable PICF illustrated in Figure 2 is a particular implementation of recently introduced universal programmable filters.^24,25^ We selected a compact 4 × 4 filter in silicon-on-insulator (SOI) platform with standard components available at photonic foundries for proof-of-concept experiments. In this section, we explain the principle and illustrate the tunable filter function of this PICF.

**Figure 2:**
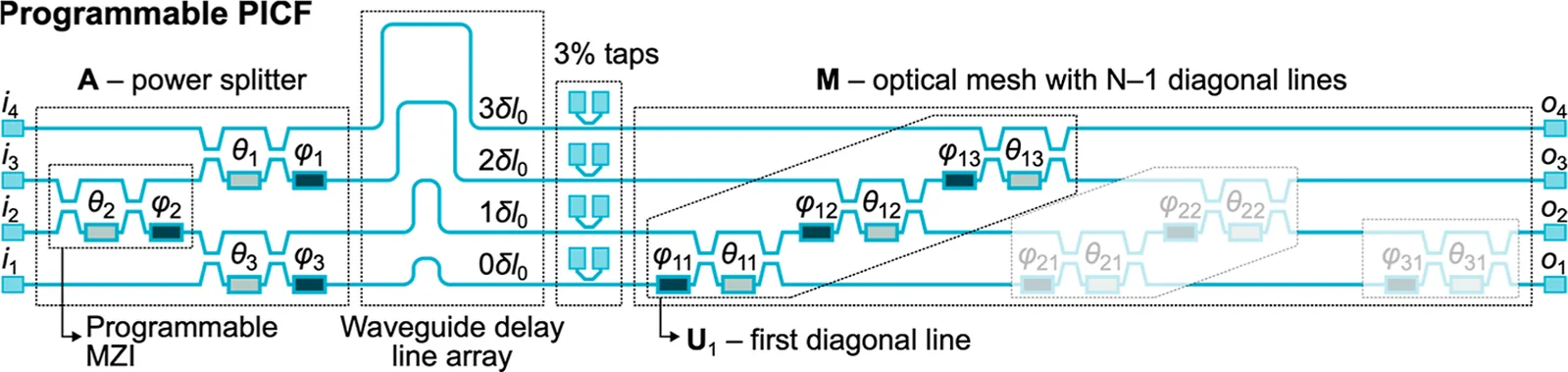
Schematics of the 4 × 4 programmable PICF. Inside the PICF, light from input *i*_3_ or *i*_2_ is split by power splitter **A** into the waveguide delay line array and then recombined in the optical mesh **M** with relative time delays. Both **A** and **M** comprise programmable MZIs, which enable control of the PICF transmission function. The delay lines are followed by 3% power taps formed by directional couplers and grating couplers. These taps are used for calibration. **M** is further divided into diagonal lines **U**_*i*_. With even power splitting in **A**, **M** can be programmed to produce functions (eq 3) at the outputs *o*_*q*_. E.g., **U**_1_ produces (3) at *o*_4_.

The PICF comprises a programmable power splitter layer as described by a transmission matrix **A**, a fixed array of waveguide delay lines, and a programmable photonic mesh layer as described by a transmission matrix **M** (throughout the paper, we denote matrices in bold capital letters). Both **A** and **M** are 4 × 4 unitary operations implemented as arrays of 2 × 2 programmable Mach–Zehnder interferometers (MZIs). Each MZI has two nominally 50:50 directional couplers and two thermal phase shifters φ and θ as shown in Figure 2, allowing them to be programmed as arbitrary unitary matrices. In other words, they can be set to any state between transparent bar state and transparent cross state

1 $$S_{bar}=\left(\begin{array}{ll} 1 & 0\\ 0 & 1 \end{array}\right)\;\text{and}{\;S}_{cross}=\left(\begin{array}{ll} 0 & 1\\ 1 & 0 \end{array}\right)$$

The power splitter **A** is a binary tree and allows arbitrary power splitting from either of the inputs *i*_2_ and *i*_3_. The transmission matrix of the optical mesh **M** is further divided into 3 unitary matrices **U**_1_, **U**_2_, and **U**_3_, corresponding to self-configuring layers that are cascaded to make the complete mesh. They are self-configuring in the sense of the MZIs being progressively configured for a given unitary operation via self-configuration algorithms,^27^ which find the desired phase shifter settings. We implement each **U**_*i*_ as a set of MZIs in a “diagonal line” self-configuring layer; the first such layer is emphasized in Figure 2, though other topologies for constructing **M** are possible.^24^

We selected a conventional waveguide delay line array with constant length increments δ*l*_0_ between the successive waveguides, i.e. δ*l*_*p*_ = (*p* – 1)­δ*l*_0_ for *p* ∈ {1, 2, 3, 4}, similarly to AWGs. An input field is split by **A** into the delay lines and time-delayed by δ*t*_*p*_ = *n*_g_δ*l*_*p*_/*c* where *n*_g_ is a group index and *c* is the velocity of light. The time-delayed fields are harmonic functions of ωδ*t*_*p*_, that is exp­{−*i*ωδ*t*_*p*_}, and they can be arbitrarily interfered or summed in the mesh **M**. Note that potential errors in the phase of the time-delayed fields coming from fabrication tolerances are corrected by the φ phase shifters during self-configuration of **M**. The sum of such harmonic fields exiting **M**, after correcting for fabrication tolerances, is always periodic because the relative time delays are integer multiples of δ*t*_0_. This period is equivalent to an FSR of the system with a frequency

2 $$f_{FSR}=\frac{c}{n_{g}\delta l_{0}}$$

or in angular frequency ω_FSR_ = 2π*f*_FSR_. This is true regardless of the power splitting in **A** and summing coefficients in **M**. However, we are particularly interested in maximizing transmission of the PICF at some frequency ω on one of the outputs *o*_*q*_ for efficient operation inside the laser cavity.

In a special case of even power splitting in **A** and with our delay line array choice δ*l*_*p*_ = (*p* – 1)­δ*l*_0_, the optimal configuration of **M** results in a “sinc-like” output function at *o*_*q*_ common in AWG systems

3 $$y_{q}=e^{-i\left(N-1\right)/2\alpha }\frac{sinc\left(N\alpha /2\right)}{sinc\left(\alpha /2\right)}x\left(\omega \right)$$

where *N* is the number of delay lines, thus *N* = 4 in our filter, *x*(ω) is the PICF input amplitude as a function of frequency, and α is a phase factor. In a passive device, α would be a simple product of frequency and time delay α = ωδ*t*_0_ with (eq 3) being a fixed function of ω. However, the programmable MZIs allow us to add a controlled linear “phase tilt” γ to the delay lines via the φ phase shifters in **A** or in **U**_1_. This means progressively adding increments of 2πγ to the phase of the fields in the delay lines, or in absolute terms (*p* – 1)­2πγ to *p*-th delay line. Such progressive phase adjustments appear as phase tilting across the delay line array, hence the term phase tilt. The phase factor expands to α = ωδ*t*_0_ – 2πγ and changing γ within an interval (0, 1) shifts (eq 3) in frequency. Values outside of this interval would be redundant due to the periodicity.

Figure 3 shows function (eq 3) for *N* = 4 delay lines, *f*_FSR_ = 100 GHz, and for different values of the linear phase tilt γ around a central wavelength of 1550 nm, which is related to angular frequency as λ = 2π*c*/ω. A delay line increment δ*l*_0_ = 740 μm was calculated from (eq 2) for *n*_g_ = 4.0567 obtained with FDTD solver EMopt for nominally 500 nm wide and 220 nm thick silicon waveguides available in the selected SOI platform. This choice further determines the bandpass width Δ*f* defined as the period of frequencies where (eq 3) is nulled

4 $$\Delta f=\frac{c}{Nn_{g}\delta l_{0}}$$

**Figure 3:**
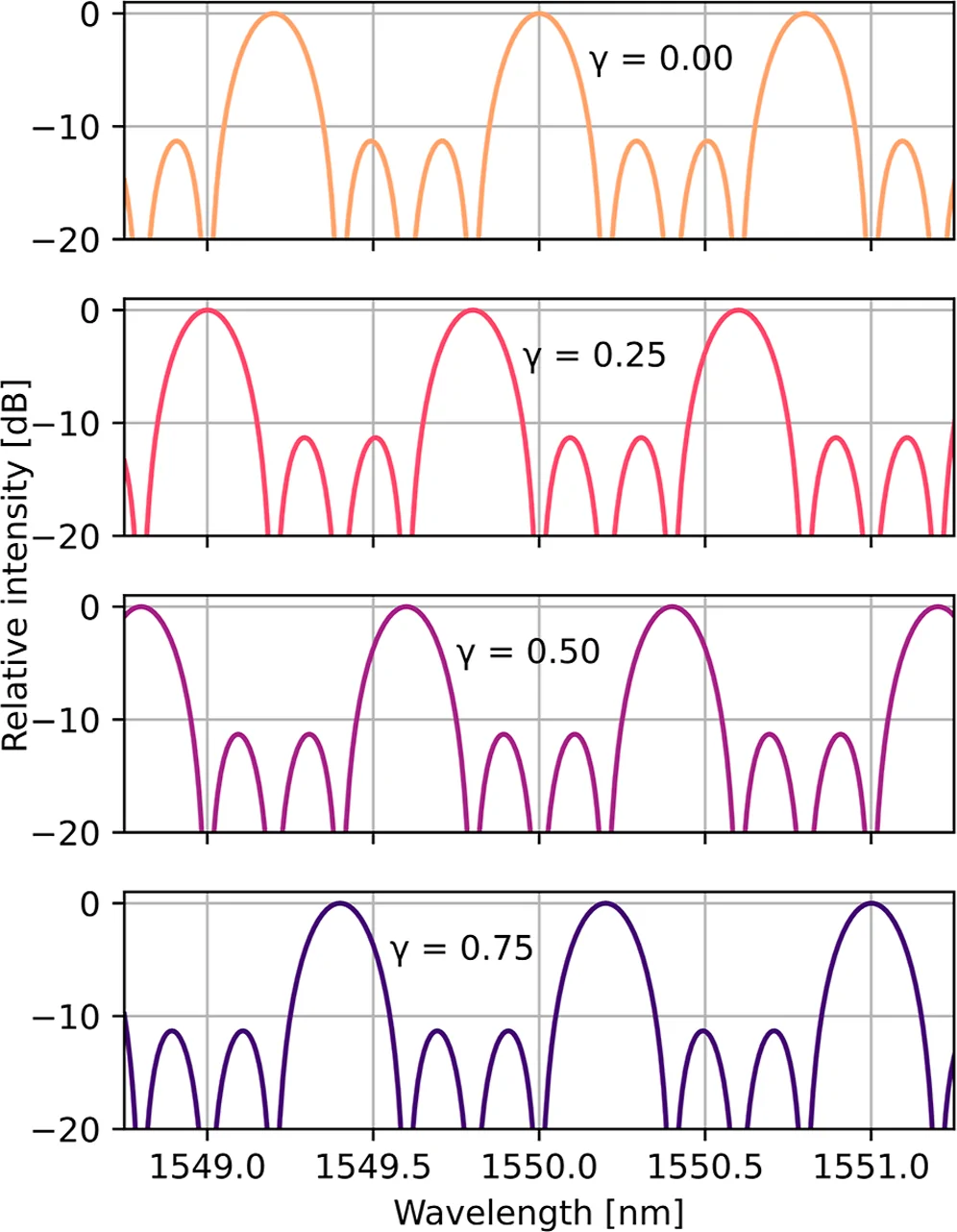
PICF filter function and tuning by the linear phase tilt γ. The filter functions profiles are calculated from (eq 3) for *N* = 4 and δ*l*_0_ = 740 μm with the phase factor α = ωδ*t*_0_ – 2πγ. The FSR of this filter function is *f*_FSR_ = 100 GHz and bandpass width Δ*f* = 25 GHz.

The resulting bandpass width of our filter is Δ*f* = 25 GHz. This *f*_FSR_ and Δ*f* correspond to ∼0.8 nm FSR and ∼0.2 nm bandpass width around 1550 nm wavelength. Further technical details of the implemented PICF are provided in the Methods section.

The filter requires initial calibration of the **A** and **M** layers in order to configure the PICF transmission to functions of form (eq 3). This procedure is described in more detail in the Methods section and in Valdez et al.^25^ First, a fixed wavelength pilot laser (1 kHz linewidth) is launched to input *i*_3_. To control the power splitting in **A**, a mapping is found between θ_1_, θ_2_, and θ_3_ heater powers and the respective phase shifts using the 3% power taps. Note that the positions of the power taps at the delay line outputs allow us to correct for propagation losses after the chip fabrication. Then, all MZIs in **M** are calibrated to set them into transparent bar states **S**_bar_ (eq 1) as if the MZIs are not there, which is necessary for initiating the self-configuration algorithms.^27^ Finally, the algorithm progressively adjusts both φ and θ phase shifters in **M** to maximize the power at the outputs *o*_*q*_ at some selected pilot laser wavelengths. E.g., **U**_1_ is self-configured to maximize the power at *o*_4_ for λ = 1550 nm while the remaining outputs of **U**_1_ become the inputs of the following diagonal line **U**_2_. Importantly, configuring φ phase shifters during the self-configuration corrects time delay errors coming from fabrication imperfections. Self-configuring **U**_1_ is sufficient for laser tuning, but other applications will require calibration of the remaining diagonal lines of **M**.

## 2 Results

### 2.1 Ring Cavity Laser Tuning with the PICF

The architecture of our test laser in ring cavity configuration is shown in Figure 4. The custom programmable PICF is controlled from a personal computer (PC) via a Digital-to-Analog Converter (DAC) and sets the emission wavelength of the laser, which uses a 30 dBm erbium-doped fiber amplifier (EDFA, IRE-Polus Group, Model EAU-1M) as the laser gain medium. The EDFA output is coupled to a 90:10 beamsplitter (BS). The 10% output is used for characterization by an optical spectrum analyzer (OSA), while the 90% output circulates back to the EDFA input through a series of optical modules: An optical circulator (OC) to ensure unidirectional lasing operation, a (fixed) 8-channel WDM device to select part of the EDFA gain bandwidth, a Fiber Polarization Controller (FPC) to match TE polarization in the PICF waveguides, and finally the PICF before reaching the EDFA. All components are fiber-pigtailed except for the PICF, which in principle can also be pigtailed, so the overall system is compact and robust without any mechanical moving structures.

**Figure 4:**
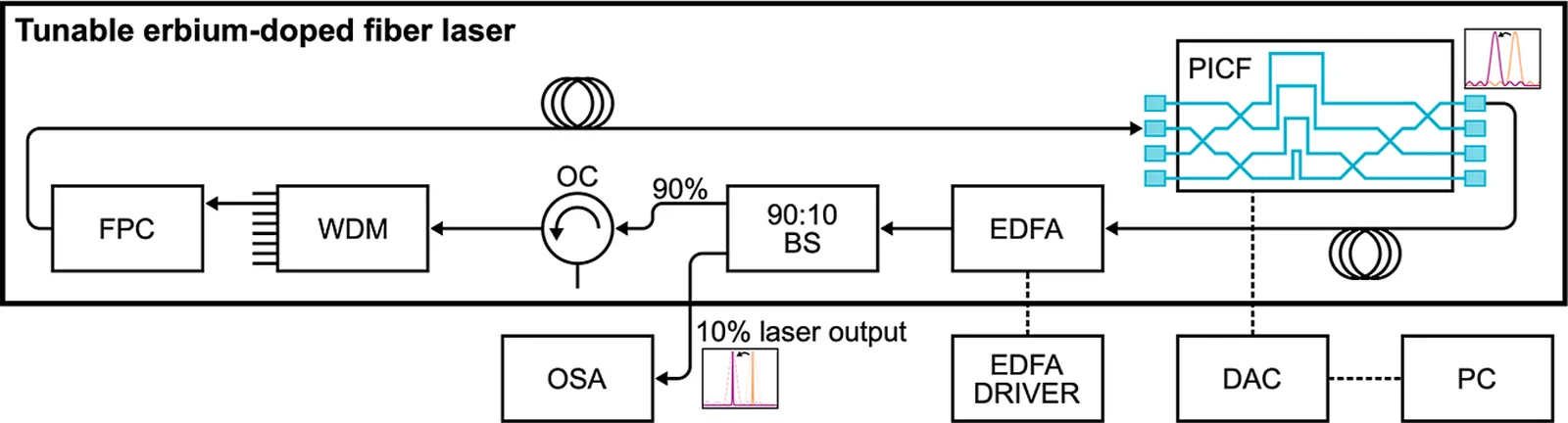
Detailed schematics of the tunable laser. EDFA—erbium-doped fiber amplifier, BS—beamsplitter, OSA—optical spectrum analyzer, OC—optical circulator, WDM—wavelength division multiplexing device (fixed, 8 channels), FPC—fiber polarization controller, DAC—digital-to-analog converter. Black solid lines represent single-mode optical fibers, blue solid lines represent single-mode optical waveguides, and dashed lines represent electrical connections. Light is coupled from the optical fiber in and out the PICF waveguides via grating coupler input *i*_3_ and output *o*_4_ respectively.

Our PICF is intentionally placed in the ring before the EDFA to prevent exceeding the damage threshold of the PICF. This way, maximum optical power is dissipated before returning to the input of the PICF. The commercial fixed flat-top WDM module with Δ*f* = 100 GHz bandpass width limits the EDFA gain bandwidth to a single 100 GHz FSR of the PICF (*f*_FSR_ = 100 GHz). Without the fixed WDM, the laser would operate simultaneously at multiple wavelengths separated by the 100 GHz FSR of the PICF. This would lead to competition for the EDFA gain and unstable power output if not adequately managed.

The laser ring cavity setup was first characterized to determine its transmission. The EDFA was operated without the PICF to obtain the broadband amplified spontaneous emission of the EDFA (Figure 5a). This emission output was separately launched into the WDM device and the programmable PICF with different γ values, and the transmission was recorded with the OSA and normalized (Figure 5b). We selected the fixed WDM channel around 1555 nm for the relatively flat EDFA gain compared to the 1550 nm channel. The measured FSR of the PICF is 94.83 GHz, about 5% lower than the nominal 100 GHz. This is likely due to a difference between the design and real *n*_g_, while the influence of the shift to 1555 nm from 1550 nm is negligible as further discussed in the next section. Figure 5c shows the product of the WDM and the PICF transmission and has the significance of the transmission profile of the ring cavity from Figure 4.

**Figure 5:**
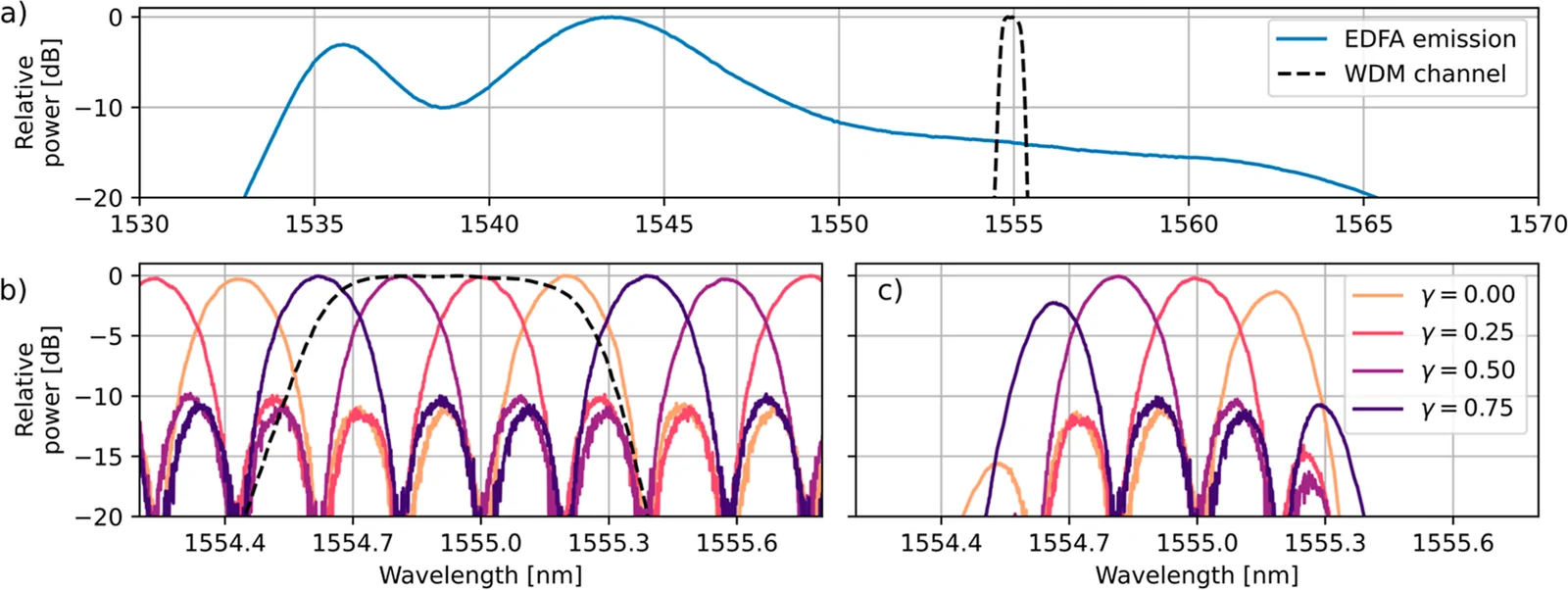
Normalized EDFA emission and ring cavity transmission functions. (a) EDFA spontaneous emission when operated at 0.8 A and the transmission of a selected channel of the commercial WDM. (b) Transmission profiles of the PICF for different phase tilt γ values shown over the WDM transmission. (c) Product of the PICF transmission curves and the WDM transmission. This is consequently the transmission profile of the laser ring cavity at different γ values.

Measured loss of the ring cavity without the EDFA was 22.8 dB, while the PICF itself accounted for 16.2 dB. The latter is dominated by 6 dB coupling loss per grating coupler. This figure is typical for grating couplers, but sub-dB coupling loss can be achieved.^28^ Similarly, inverse taper edge couplers^29^ have shown 0.7 dB loss^30^ or even less than 0.25 dB with mode converters.^31^ Waveguides, on the other hand, contribute only up to ∼3 dB with up to 3 dB cm^–1^ propagation loss and ∼7–10 mm length. The remainder is attributed to directional coupler excess loss and optical fiber connectors. Despite the large total losses of the ring cavity, it is possible to operate the fiber laser because the saturated amplification of the EDFA is as high as 30 dB.

The ring cavity laser experiments used a 0.5 A pump current to the EDFA module, well below the maximum current of 2 A. This kept the circulating optical power low to avoid damage to the PICF or nonlinear effects. Figure 6 shows the laser emission from the 10% tap recorded with the OSA indicating output power up to ∼25 mW. The emission wavelength follows the filter function; As γ is changed from 0 to 0.75, the wavelength shifts down from 1555.19 to 1554.66 nm. Adjusting further to γ = 1 would result in aliasing the output back to the position of γ = 0, that is to 1555.19 nm. In total, the laser can be tuned by ∼0.6 nm, slightly less than the ∼0.8 nm FSR of the PICF. This limit is imposed by the transmission profile of the WDM channel, which is necessary for single-wavelength laser operation.

**Figure 6:**
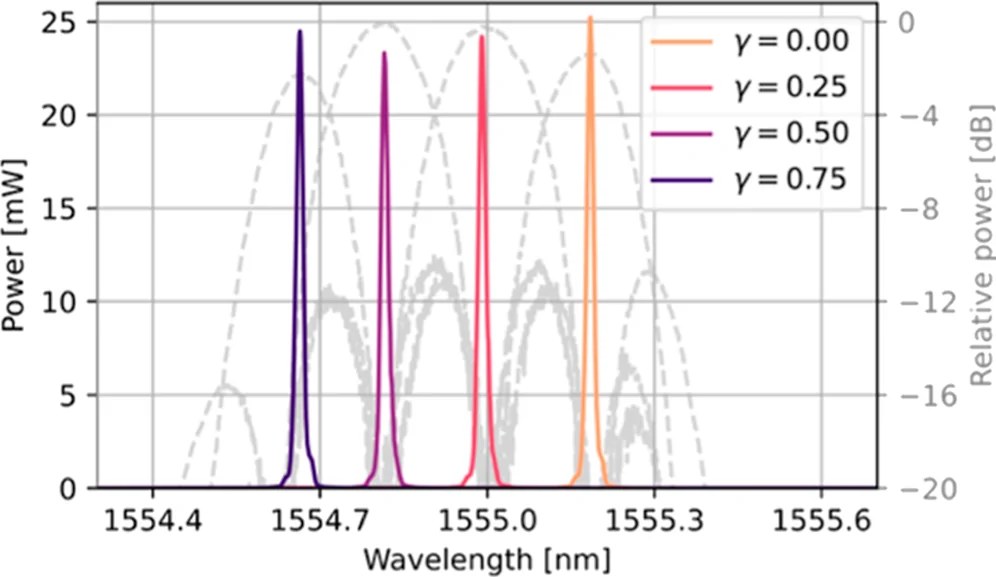
Laser emission wavelength tuning in free-running regime using the programmable PICF. The laser emission line tracks the position of the laser cavity transmission given by the WDM and the PICF (dashed gray curves) as γ is tuned from 0 to 0.75.

The linewidth obtained with the OSA shows about ∼2 GHz. This value is limited by the OSA’s monochromator 0.02 nm resolution (∼2 GHz around 1555 nm), which indicates that ∼2 GHz is the linewidth upper limit. However, a frequency instability could be observed with the OSA, which is likely due to the large bandpass width (25 GHz) of our PICF combined with the high round-trip loss. This complicates heterodyne measurements against the 1 kHz pilot laser, which did not provide useful measurements of the linewidth.

### 2.2 Dispersive Model of the 4 × 4 Programmable PICF

The analytical PICF model of eq 3 needs refinement to investigate broader tuning characteristics of the ring laser cavity, so we develop a numerical model that accounts for wavelength dependence of the individual components. The waveguide delay line array dispersion and the directional couplers dispersion within the MZIs are modeled with wavelength-dependent effective indices found with Lumerical MODE using Si and SiO_2_ dispersion data. The dispersion of the thermal phase shifters was modeled with Lumerical HEAT and MODE solvers to find the temperature distribution and modal effective index subject to thermo-optic coefficients of Si and SiO_2_. Lumerical MODE yields a larger group index *n*_g_ = 4.243 at 1550 nm (*n*_g_ = 4.2438 at 1555 nm) compared to the EMopt solver giving *n*_g_ = 4.0567 at 1550 nm. This yields an FSR of 95.48 GHz, lower from the nominal 100 GHz that the current PICF is based on, and closer to the experimental FSR.

The dispersive model is centered at 1555 nm to match with the laser emission wavelength, and the model output is obtained after optimizing **A** for even power splitting and the first diagonal line **U**_1_ for maximum transmission at 1555 nm. In addition to the PICF, the dispersion of the grating couplers is accounted for. We model the grating couplers as having a Gaussian wavelength dependence with a 1 dB bandwidth of 20 nm centered at 1555 nm.^28^

Figure 7a shows the dispersive model results including the passing of two grating couplers to account for in-coupling and out-coupling. The coupling loss of about 12 dB is omitted to highlight the dispersion effects. This result is nearly identical to the analytical model from Section 2 apart from the FSR, and shows more than 10 dB rejection out of the 25 GHz bandpass. This rejection would be further increased with more delay lines in the filter. The power transmission modulation is negligible across several FSRs around 1555 nm, but it drops quickly in a wider bandwidth of tens of nanometers due to the grating couplers (Figure 7b), with a 1 dB bandwidth of ∼14 nm. However, the bandwidth can be extended with edge couplers or on-chip integration with the gain medium. Without the grating couplers, the model predicts the maximum transmission decreasing by only 0.05 dB after detuning by 20 nm, which is hardly visible in Figure 7b. The device can be thus readily operated across more than the C-band with standard silicon photonic components.

**Figure 7:**
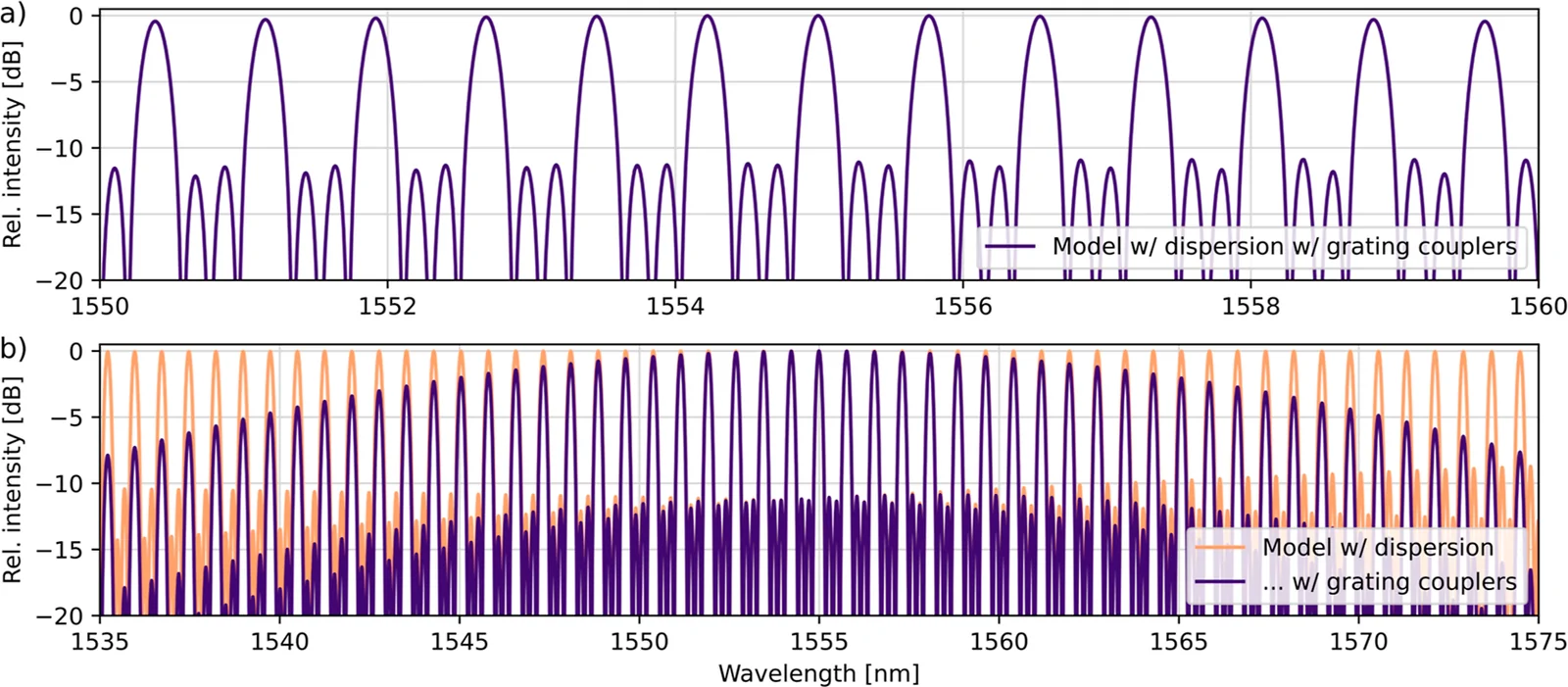
A dispersive model of the PICF transmission centered at 1555 nm. (a) Dispersive model transmission accounting for dispersion in the delay line array, directional couplers, thermal phase shifters, and grating coupler dispersion. The overall 12 dB coupling loss is omitted. (b) Expanded view of (a) over 40 nm bandwidth, adding dispersive model results without the influence of grating couplers dispersion.

## 3 Discussion

The presented proof-of-concept programmable PICF demonstrates the utility of forward-only interferometer meshes in laser wavelength tuning. By controlling the phase tilt γ in the waveguide delay line array, we can shift the emission wavelength by up to ∼0.6 nm. In principle, we could shift the single-wavelength output by the full FSR of 0.8 nm or 100 GHz, but the current system is limited by the transmission of the additional fixed WDM. Such an additional filter is required for gain media with bandwidths larger than 0.8 nm such as the erbium-doped fiber. Nevertheless, the demonstrated tuning range is already applicable to, e.g., gas spectroscopy, which can be performed in a narrow wavelength range.^2,5^

Emission linewidth measurements on the OSA with 0.02 nm/2 GHz resolution suggest an upper limit of 2 GHz with an observable frequency jitter due to the relatively broad PICF bandpass width Δ*f* = 25 GHz (0.2 nm) and high roundtrip loss. Hence, lower optical loss, larger FSR, and narrower bandpass function are essential to advance such PICFs. Losses can be addressed with different coupler designs and integration with gain media discussed lower, whereas the waveguide delay line array can be scaled to manipulate the FSR and bandpass function. Arrays with constant length increments, such as the one here with *N* = 4, do not scale favorably. E.g., the C-band can be covered with >4.5 THz FSR with δ*l*_0_ = 16 μm and *N* = 16, but this results in Δ*f* > 250 GHz. However, more suitable arrays exist, but are not common in waveguide delay line array design. We have previously discussed a nonredundant delay line array of the Golomb ruler type,^24^ which yields Δ*f* = ∼18 GHz for the same δ*l*_0_ = 16 μm and *N* = 16, while maintaining the FSR. This type of array takes, more generally, delay line lengths δ*l*_*p*_ = *m*_*p*_δ*l*_0_ with integers *m*_*p*_ taken from a 16th order Golomb ruler set.

The PICF leverages the reliability of silicon photonics at wavelengths around 1550 nm, making EDFA a perfect and available choice for the tunable laser demonstrator. Nonetheless, it can be adapted to different emission wavelengths. Other rare earth elements like ytterbium, or holmium in their suitable host glass fibers enable operation from ∼500 nm to 3 μm, with bandwidths of 10s of nanometers to over 100 nm.^32,33^ Even more appealing is the development of thin films doped with active ions for amplifiers and lasers with platforms like Y:Ta_2_O_5_,^34^ Er:Si_3_N_4_,^35^ or Er:Al_2_O_3_ ^36^ that could heterogeneously integrate with our PICF. A single PIC-based Ti:Sa laser with gain bandwidth over 400 nm has already been demonstrated to emit across more than 50 nm with a single-wavelength output.^37^ Semiconductor gain media present another opportunity, offering bandwidths of 10s of nanometers to over 100 nm and cover wavelengths from UV to mid-infrared through bandgap engineering of III–V semiconductors.

Accessing different wavelengths and larger bandwidths with this PICF concept requires adapting the dimensions of the nanophotonic components and even changing material platform. Short wavelengths below the Si transparency cutoff around 1.1 μm can be covered with Si_3_N_4_.^21^ Long wavelengths beyond 3.5 μm, where the SOI is limited by the SiO_2_ absorption, can be processed with germanium platforms^38^ or suspended silicon.^39^ The operational bandwidth and attainable output power are mainly driven by the grating couplers with a nominal 1 dB bandwidth of 20 nm and coupling loss as much as 6 dB, requiring an odd 80° coupling angle. Novel grating couplers based on three-wave interaction surmount these drawbacks, showing 1 dB bandwidth of 20 nm and 0.69 dB coupling loss at normal incidence,^28^ easing the optical alignment. In theory, such gratings can achieve 8 nm bandwidth with 0.25 dB coupling loss or 48 nm/0.48 dB.^40^ Edge couplers extend the 1 dB bandwidth to around 100 nm and reduce the coupling loss to 0.25 dB when mode converters are employed.^31^ Surprisingly, the conventional dispersive waveguides and MZIs do not significantly affect the performance as shown by the dispersive model results in Figure 7, losing only 0.05 dB power after detuning by 20 nm with negligible frequency drift.

PIC-based tuning mechanisms offer higher tuning speeds and compactness compared to free-space optics, e.g. moving gratings in traditional Littrow and Littman–Metcalf configurations. The forward-only PICF in this paper is a new alternative to other PIC-based mechanisms like Vernier tuning with a pair of microresonators^13,21^ or DBRs.^12^ These require simultaneous sweeping of resonances for continuous wavelength tuning, and rematching of resonances to cover large bandwidths. In the forward-only system, the wavelength tuning can be fully facilitated with the phase tilt γ, or we can switch the configuration of the mesh **M**. In addition, we can leverage other diagonal lines to simultaneously control multiple wavelengths. Presently, the PICF is based on thermal phase shifters, which can switch between configurations with a 30 μs time constant,^25^ that is with speeds over 10 kHz. Even higher speeds will be possible with MEMS or electro-optic phase shifters.

## 4 Conclusion

We demonstrated a laser wavelength tuning with an intracavity solid-state 4 × 4 PICF with 25 GHz bandpass width and 100 GHz FSR. The filter is based on forward-only optical meshes and is fabricated with only standard silicon photonics components in a photonic foundry. First mesh **A** facilitates arbitrary power splitting, a delay line array with *N* = 4 waveguides introduces time delays, and a second mesh **M** interferes the time-delayed complex fields to generate transmission functions such as (eq 3). The laser ring cavity comprises fiber-optic and pigtailed components, which are readily interfaced with the PICF through grating couplers. We continuously tuned the laser emission wavelength by ∼0.6 nm around a central wavelength of 1555 nm with output power up to 25 mW. This tuning range is intentionally restricted by a fixed flat-top WDM device in the laser ring cavity. The WDM has Δ*f* = 100 GHz matching the PICF FSR and thus ensuring single-wavelength operation.

The 0.6 nm tuning is applicable to e.g., gas spectroscopy, but the 25 GHz bandpass needs to be narrowed down. This can be achieved by increasing the number of channels in the waveguide delay line array and abandoning the current uniform array design in favor of nonredundant ones such as Golomb rulers. Simultaneously, the FSR can extend beyond the gain bandwidth using shorter delay increments δ*l*_0_. Moreover, a significant increase in power efficiency is expected by implementing couplers with losses around 1 dB, or by integrating with gain media. This PICF concept will extend the device library for photonics engineers and aid addressing the needs for compact broadly tunable lasers. Finally, it offers filter function engineering and potentially enables independent control of multiple lasing wavelengths, neither of which is available with microresonator architectures.

## 5 Methods

### 5.1 Analytical Derivation of the Filter Response

We consider a PICF with the architecture in Figure 2 but generalized to *N* × *N*, that is with *N* waveguide delay lines with constant length increments δ*l*_0_ between the successive waveguides, i.e., δ*l*_*p*_ = (*p* – 1)­δ*l*_0_ for *p* ∈ {1, ..., *N*}. Every waveguide of such delay line array outputs a complex optical field with a phase shift that is a multiple of the time delay

M1 $${\delta t}_{0}=n_{g}{\delta l}_{0}/c$$

where *n*_g_ is a group index, and *c* is the velocity of light. Considering the frequency as δω = (ω – ω_*c*_), relative to a central frequency ω_*c*_, we write the field leaving waveguide delay line *p* as

M2 $$E_{p}=a_{p}\exp\left\{-i\left(p-1\right)\delta \omega {\delta t}_{0}\right\}x\left(\delta \omega \right)$$

where *a*_*p*_ is an amplitude fraction imposed by the power splitter **A** and input field *x*(δω). We further add the programmable linear phase tilt γ implemented with the φ phase shifters in **A** or **U**_1_. The additional phase in the *p*-th delay line is δ_*p*_ = 2π­(*p* – 1)­γ with γ ∈ (0,1], effectively progressively changing the lengths of the delay lines. We can rewrite (M2) with γ as

M3 $$E_{p}=a_{p}\exp\left\{-i\left(p-1\right)\left(\delta \omega {\delta t}_{0}-2\pi \gamma \right)\right\}x\left(\delta \omega \right)$$

The outputs *o*_*q*_ will produce superpositions of such time-delayed fields from the waveguide delay line array

M4 $$y_{q}=x\left(\delta \omega \right)\sum_{p=1}^{N}M_{qp}a_{p}\exp\left\{-i\left(p-1\right)\left(\delta \omega {\delta t}_{0}-2\pi \gamma \right)\right\}$$

where *M*_*qp*_ are elements of the unitary matrix **M** describing the optical interferometer mesh. This sum representing the filter response is periodic in frequency since the harmonic terms are the same at δωδ*t*_0_ = 2*n*π for any integer *n*. The period 2π/δ*t*_0_ is the FSR of the device and, using (M1), we get

M5 $$\omega_{FSR}=\frac{2\pi c}{n_{g}\delta l_{0}}\;\text{or}\;f_{FSR}=\frac{c}{n_{g}\delta l_{0}}$$

We further assume even power splitting in **A** for simplicity, i.e. $a_{p}=1/\sqrt{N}$ for all *p*, and uniform matrix **M** elements, which means $M_{qp}=1/\sqrt{N}$ since **M** is unitary. Then the sum in (M4) can be carried out to yield the solution

M6 $$y_{q}=e^{-i\frac{\left(N-1\right)}{2}\left(\delta \omega {\delta t}_{0}-2\pi \gamma \right)}\frac{sinc\left(N\left[\delta \omega \delta t_{0}-2\pi \gamma \right]/2\right)}{sinc\left(\left[\delta \omega \delta t_{0}-2\pi \gamma \right]/2\right)}x\left(\delta \omega \right)$$

For γ = 0, such a function is exactly one at δω = 0 and zero for a set of frequencies δω_*s*_ = 2π*s*/*N*δ*t*_0_ for *s* ∈ {1, 2, ... *N* – 1}. The period of the frequencies δω_*s*_ is the filter bandpass width and reads

M7 $$\Delta \omega =\frac{2\pi c}{Νn_{g}\delta l_{0}}\;\text{or}\;\Delta f=\frac{c}{Nn_{g}\delta l_{0}}$$

### 5.2 Implementation of the PICF Chip

The 4 × 4 PICF is designed using SOI waveguides with nominally 500 nm wide and 220 nm high Si core cladded by SiO_2_ for 100 GHz FSR and bandpass width Δ*f* = 25 GHz around a central wavelength of 1550 nm. These figures correspond to ∼0.8 nm FSR and ∼0.2 nm bandpass width. For *N* = 4 waveguides in the delay line array and a group index *n*_g_ = 4.0567 obtained with FDTD solver EMopt, a required delay line increment δ*l*_0_ = 740 μm is calculated from (eq 2) (also (M5)).

Each MZI in **A** and **M** has two 40 μm long directional couplers with a 300 nm gap for nominally 50:50 power splitting, and two 120 μm long thermo-optic phase shifters φ and θ.^41^ The heaters are thermally isolated by deep-etched trenches to minimize thermal cross-talk. Coupling between optical fibers and the waveguides is facilitated by standard SOI grating couplers.

Finally, the PICF chip was fabricated in the SOI platform in a multiproject wafer run at advanced micro foundry (AMF). The fabricated chip was then attached and wire-bonded to a custom printed circuit board to interface the phase shifters and the digital-to-analog converter and controlled from a PC.

### 5.3 PICF Calibration

The PICF is calibrated in 3 major steps: (1) map the θ phase shifts in the power splitter **A** to the supplied heater powers; (2) find the bar states **S**_bar_ (eq 1) of MZIs in **M**, which is a prerequisite for the last step; (3) set **M** to the desired unitary operation by self-configuration algorithms.^27^

1 Calibrate **A**. To calibrate θ_1_, θ_2_, and θ_3_ in **A**, a fixed wavelength pilot laser (1 kHz linewidth) is launched to input *i*_3_. The θ_1_ phase shifter power is swept while monitoring the power from the 3% taps on the delay lines *p* = 3 and 4 and this mapping is stored. Phase shifter θ_3_ is calibrated with taps on lines 1 and 2, while θ_2_ requires monitoring the sums from taps on lines 1 + 2 and 3 + 4. Once the mapping between the electrical powers and phase shifts is found, **A** can be set to even power splitting, or to other power distributions. Note that the positions of the power taps at the delay line outputs allow us to correct for propagation losses after the chip fabrication.
2 Calibrate **M**. The θ_*ij*_ phase shifters in **M** are calibrated by setting **A** to send all power from input *i*_3_ to delay line *p* = 1. First, the phase shifter θ_13_ heater power is swept while monitoring the power at output *o*_4_. θ_13_ is then set to a cross state **S**_cross_ (eq 1), transmitting light from the bottom input of the MZI to the top output. Phase shifter θ_12_ is then calibrated while again monitoring *o*_4_ and set to a cross state. The procedure is finally repeated for θ_11_, completing the calibration of the first diagonal line **U**_1_. All MZIs in **U**_1_ are then set to a transparent bar state **S**_bar_ and the calibration procedure repeats with phase shifters θ_2*j*_ in **U**_2_ and so on until the whole **M** is calibrated.
3 Self-configure **M** to the desired unitary operation. The power splitter **A** is set to even splitting and the mesh **M** is set to a transparent bar state to initiate the self-configuration algorithm.^27^ Starting with **U**_1_, this algorithm progressively adjusts both θ and φ phase shifters of each MZI in diagonal line **U**_*i*_ to collect all light from a pilot laser at frequency ω_*i*_ in a single output *o*_*N*–*i*+1_. E.g., **U**_1_ is optimized for collecting light in *o*_4_ while the pilot laser is launched into *i*_3_. Since we are concerned with tuning a single wavelength emitted by a laser, we can reduce the calibration of **M** described here to calibration of **U**_1_. Complete **M** calibration may be required for other filter applications.

## Abbreviations

AWG: arrayed waveguide grating
BS: beamsplitter
DAC: digital-to-analog converter
DBR: distributed Bragg reflector
EDFA: erbium-doped fiber amplifier
FPC: fiber polarization controller
FSR: free spectral range
MZI: Mach–Zehnder interferometer
OC: optical circulator
OSA: optical spectrum analyzer
PIC: photonic integrated circuit
PICF: PIC filter
SOI: silicon on insulator
WDM: wavelength division multiplexing
